# Long-Term Functional Outcomes Following Surgical Treatment of Spinal Schwannomas: A Population-Based Cohort Study

**DOI:** 10.3390/cancers16030519

**Published:** 2024-01-25

**Authors:** Aman Singh, Alexander Fletcher-Sandersjöö, Victor Gabriel El-Hajj, Gustav Burström, Erik Edström, Adrian Elmi-Terander

**Affiliations:** 1 Department of Clinical Neuroscience, Karolinska Institute, 171 76 Stockholm, Sweden; aman.gill.singh99@gmail.com (A.S.); alexander.fletcher-sandersjoo@ki.se (A.F.-S.); gustav.burstrom@ki.se (G.B.); erik.edstrom.1@ki.se (E.E.); 2 Department of Neurosurgery, Karolinska University Hospital, 171 76 Stockholm, Sweden; 3 Capio Spine Center Stockholm, Löwenströmska Hospital, 194 89 Upplands-Väsby, Sweden; 4 Department of Medical Sciences, Örebro University, 701 82 Örebro, Sweden; 5 Department of Surgical Sciences, Uppsala University, 752 36 Uppsala, Sweden

**Keywords:** schwannoma, spine, spinal schwannoma, neurosurgery

## Abstract

**Simple Summary:**

Spinal schwannomas are the second most common form of primary intradural spinal tumor. Clinically and histologically, they are classified as nerve sheath tumors. These tumors may give rise to spinal cord compression with acute or chronic neurologic dysfunction. The primary treatment is surgical resection. In this population-based cohort study, we retrospectively reviewed 180 cases of surgically treated spinal schwannomas to assess postoperative complications and long-term clinical and radiological outcomes, as well as progression-free survival. The median follow-up time was 4.4 years. Significant neurological improvement was seen following surgical treatment. Gross total resection was achieved in 83% of cases and was associated with a higher chance of progression-free survival.

**Abstract:**

Spinal schwannomas are the second most common form of primary intradural spinal tumor. Despite being benign, they may cause spinal cord compression and subsequently acute or chronic neurological dysfunction. The primary treatment is surgical resection. The aim of this study was to identify pre- and postoperative predictors of favorable outcomes after surgical treatment for spinal schwannoma. All adult patients surgically treated for spinal schwannoma between 2006 and 2020 were eligible for inclusion. Medical records and imaging data were retrospectively reviewed. The primary outcome measures were neurological improvement according to the modified McCormick Scale (mMC) and changes in motor deficit, sensory deficit, gait disturbance, bladder dysfunction, and pain at long-term follow-up. In total, 180 patients with a median follow-up time of 4.4 years were included. Pain was the most common presenting symptom (87%). The median time between symptom presentation and surgery was 12 months, while the median time between diagnosis (first MRI) and surgery was 3 months. Gross total resection (GTR) was achieved in 150 (83%) patients and the nerve root could be preserved in 133 (74%) patients. A postoperative complication occurred in 10 patients (5.6%). There were significant postoperative improvements in terms of motor, sensory, gait, and bladder functions, as well as pain (*p* < 0.001). Of these symptoms, bladder dysfunction was the one most often improved, with complete symptom resolution in all cases. However, no other predictors of improvement could be identified. There were three cases of recurrence after GTR and nine cases of regrowth after STR. Reoperation was performed in six (3.3%) cases. GTR was associated with a significant improvement in neurological status at long-term follow-up and increased the chance of progression-free survival.

## 1. Introduction

Schwannomas are benign (World Health Organization grade I), typically encapsulated nerve sheath tumors composed of well-differentiated Schwann cells [1,2]. Malignant Schwann cell-derived tumors are rare and classified as malignant nerve sheath tumors (MNST) according to the WHO classification of 2016 [3,4]. Spinal schwannomas account for approximately 25% of all intradural spinal cord tumors [3,5,6,7], with an incidence of 0.3–0.7 per 100,000 person years [8]. Tumors may be both intra- and extradural and may extend through the neural foramen to develop an hourglass or dumbbell shape [9]. Through nerve root or spinal cord compression, tumors may cause both sensory and motor dysfunction and are often associated with radicular pain [7,9,10,11]. The treatment of choice for symptomatic spinal schwannomas is surgery, aiming for gross total resection (GTR) with preservation of the nerve and sustained or improved neurological function [3,9,12]. Surgical complications often occur in large tumors with extraforaminal extension since they require more extensive approaches [13]. Intraoperative neurophysiological monitoring may be beneficial in certain cases [3].

Several studies have shown that GTR can be achieved with sustained or improved neurological function, thereby reducing the recurrence rate [3,9,12]. According to the literature, GTR is achieved in 74–94% of spinal schwannomas and subtotal resection (STR) in 15–21% of cases [2,3,6,14]. Tumor recurrence despite GTR is reported in 4–9% of cases and regrowth following STR in up to 30% of cases [3,15,16]. Postoperative neurological deficits are reported in 13–27% of cases, where subtotal resection is associated with greater postoperative motor and sensory deficits [2,6]. At postoperative follow-up, around 60% of the patients have a complete resolution of pain, while 64% with immediate postoperative neurological deficits fully recover after 12 months [6].

In this population-based cohort study, we retrospectively reviewed 180 cases of surgically treated spinal schwannomas, assessing baseline data, postoperative complications, long-term clinical and radiological outcomes, and possible predictors of postoperative neurological improvement.

## 2. Materials and Methods

### 2.1. Patient Selection and Study Setting

All adult patients surgically treated for a spinal schwannoma between 2006 and 2020 were eligible for inclusion. Patients with preexisting neurological deficits were not excluded. Patients with neurofibromatosis I/II and those lost to long-term follow-up were excluded. The study hospital is a publicly funded and owned tertiary care center that serves a region of about 2.3 million inhabitants with neurosurgical care [17]. The study was approved by the Regional and National Ethical Review Board (Dnr: 2016/1708-31/4 and 2021-05249).

### 2.2. Surgical Technique and Follow-Up Routine

Prior to surgery, the spinous process of the vertebra adjacent to the tumor (if thoracic or lumbar) was identified using computed tomography guidance and marked with the injection of a sterile carbon suspension. For cervical tumors, levels were identified with fluoroscopy. Laminectomy was conducted using an ultrasonic bone scalpel (from 2012 to 2017) (Misonix Inc., Farmingdale, NY, USA) or a high-speed drill with a diamond-coated bur and Kerrison rongeurs (from 2005 to 2011). Under the microscope, tumor dissection was started extradurally in dumbbell-shaped tumors with an extradural component. The dura was then incised and held open by sutures for tumor exposure. The arachnoid was dissected sharply, and the cranial and caudal poles of the tumor were identified. The tumor was then coagulated to reduce the size and blood supply and dissected from the corresponding nerve root(s). When possible, the tumor capsule was incised along the longitudinal axis of the nerve. After stepwise incisions and dissection through the layers of the tumor capsule, the tumor was enucleated and removed from the nerve. The capsule, derived from the outer connective tissue layers of the nerve (epineurium), was then sutured. Alternatively, when the nerve could not be distinguished from the tumor, a complete transection of the nerve was performed at the tumor poles. Duraplasty was performed in cases of a dumbbell-shaped tumor. Watertight dural closure was performed in all cases. When laminoplasty was performed, the laminae were repositioned using microplates (CMF Medicon Surgical Inc., Jacksonville, FL, USA). The soft tissue was then sutured in layers to close the wound. Neurophysiological monitoring was only used in 12 cases.

The institutional follow-up routine consisted of a clinical assessment 3–6 months after surgery at the outpatient department, as well as magnetic resonance imaging (MRI) 3 months and 1 year after surgery. An extended radiological follow-up was performed when clinically indicated.

### 2.3. Variables

The following pre- and postoperative data were collected: age, sex, prior spinal radiotherapy, prior spinal surgery, symptoms duration, time from diagnosis (first MRI) to surgery, laminectomy range, use of intraoperative neurophysiological monitoring, GTR/STR, preserved nerve root, tumor location, adjuvant treatment, postoperative complications (within 30 days), follow-up time, mortality, and cause of death.

The outcomes assessed were modified McCormick Scale (mMC) [18] (Table 1), motor deficit, sensory deficit, gait disturbance, bladder dysfunction, and pain. Tumor growth and recurrence were defined as a radiological growth of a tumor remnant following STR or the appearance of a new tumor after GTR.

### 2.4. Outcomes

The primary outcome measures were neurological improvement according to the modified McCormick Scale (mMC) and changes in motor deficit, sensory deficit, gait disturbance, bladder dysfunction, and pain at long-term follow-up. The secondary outcome was tumor control, analyzed as tumor growth or recurrence on postoperative MR.

### 2.5. Statistics

The Shapiro–Wilk test was used to evaluate the normality of the data. Since all the continuous data significantly deviated from a normal distribution pattern (Shapiro–Wilk test *p*-value < 0.05), they are presented as medians (ranges) and categorical data as numbers (proportions). Progression-free survival was evaluated using Kaplan–Meier curves. To determine if surgery was associated with a significant improvement in neurological status, the Wilcoxon matched-pairs signed-rank test was used. A univariable logistic regression model was used to identify predictors of an improved postoperative modified McCormick Scale (mMC). All analyses were conducted using the statistical software program R version 4.0.5. Statistical significance was set at *p* < 0.05.

## 3. Results

### 3.1. Baseline and Treatment Data

In total, 237 patients fulfilled the inclusion criteria. Of these, 57 were excluded due to missing follow-up data (n = 29), neurofibromatosis I/II (n = 16), and extraspinal tumors (n = 12). The remaining 180 patients were included in the study. The median age was 53 years and 90 (50%) were male. The most common presenting symptom was pain (n = 156, 87%), followed by sensory deficit (n = 76, 42%) and motor deficit (n = 74, 41%). The most common tumor location was the lumbosacral spine (n = 81, 45%) and 146 (82%) of the tumors were strictly intradural (Table 2).

The median time between symptom debut and surgery was 12 months, while the median time between diagnosis (first MRI) and surgery was 3 months. GTR was achieved in 150 (83%) patients and the nerve root could be preserved in 133 (74%) patients. Laminoplasty was performed in 83 (46%) cases. The most common postoperative complications were CSF leak requiring surgery in five patients (2.8%), followed by wound infection requiring antibiotics in four patients (2.2%). No adjuvant radiotherapy was offered to the patients who underwent STR (Table 3).

### 3.2. Tumor Control

**Figure 1:**
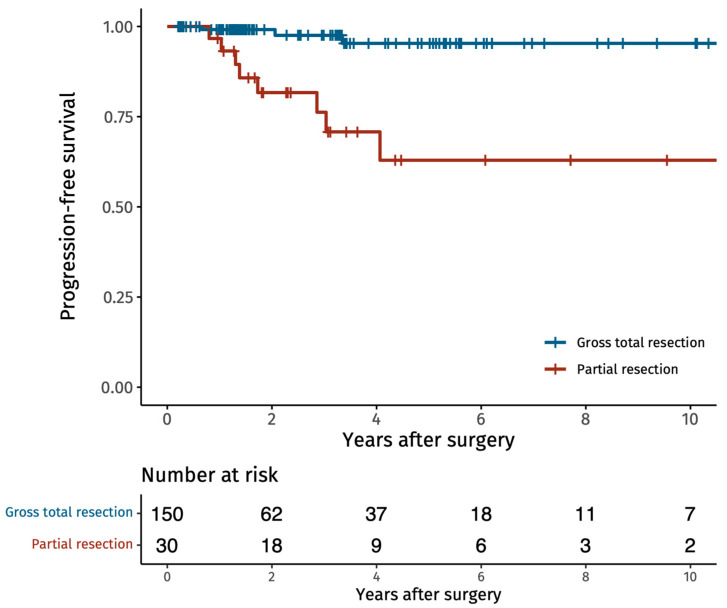
Kaplan–Meier survival curve of tumor recurrence or growth following gross total resection (GTR) or subtotal resection (STR) of spinal schwannomas. Every vertical line is when the follow-up stops for one patient.

The Kaplan–Meier survival curves show that patients undergoing GTR have a higher chance of progression-free survival after ten years compared to those undergoing STR (Figure 1).

Local tumor recurrence following GTR occurred in 3/150 (2.0%) patients, of whom 1 patient required renewed surgery. In comparison, 9/30 (30%) of the STR cases had a local tumor growth, with 5 patients requiring renewed surgery. The long-term mortality of the entire cohort was 7.8%, with no death attributed to the tumor or surgery. The median radiological follow-up time was 1.7 years and clinical follow-up time was 4.4 years (Table 4).

### 3.3. Functional Outcomes

**Figure 2:**
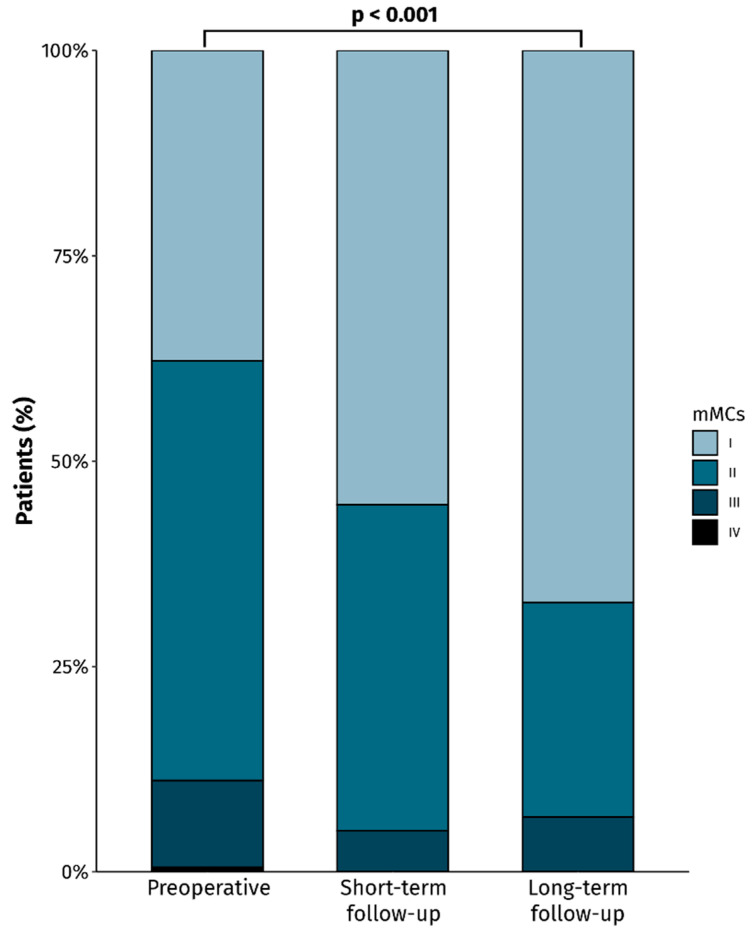
Bar chart showing the mMC scores prior to surgery and at short- and long-term follow-up of patients that underwent surgery for spinal schwannomas.

There were significant postoperative improvements in terms of motor, sensory, gait, and bladder functions, as well as pain (*p* < 0.001). Of these symptoms, bladder dysfunction was the one most often improved (complete symptom resolution in 100%), followed by gait disturbance and motor deficit (complete resolution in 91%) (Table 5). Seventy patients (39%) experienced postoperative improvements in terms of the mMC and ninety-seven patients (54%) remained unchanged (Figure 2). However, 13 (7%) patients who were mMC I prior to surgery experienced postoperative deterioration to mMC II.

### 3.4. Predictors of Improved Functional Outcome

In the univariable logistic regression predicting postoperative improvement in mMCs for those who were mMCs > I, preoperatively, no significant predictors of postoperative improvement were identified (Table 6).

## 4. Discussion

In line with previously published data, there were significant long-term postoperative improvements in neurological function in this study cohort of 180 patients with spinal schwannomas [2,6]. However, aside from surgery itself, univariate logistic regression did not identify any predictor of postoperative improvement in the mMC. Similarly, Zou et al. reported that age, sex, and the duration of symptoms were not correlated to postoperative neurological deficits [19]. However, in this study cohort, 89% of the patients had an mMC of I or II, reflecting no or only mild preoperative deficits, leaving little room for postoperative improvement.

Previous studies on extramedullary intradural tumors have been ambiguous regarding the relationship between the degree of spinal cord compression and outcomes. Some studies report that spinal cord compression and tumor area could predict improvement in functional outcomes [20,21], while others, in line with the current study, find no significant correlation [22,23]. Importantly, since neurological symptoms are mainly reflective of nerve root or spinal cord compression, only patients with symptomatic compression can improve. In line with this, authors such as Apostolov have highlighted the importance of complete tumor resection to promote full neurological recovery [12]. There is a consensus that GTR can be safely and effectively achieved for most nerve sheath tumors and that GTR reduces the risk of tumor recurrence [3,9,12,24,25]. In our cohort, GTR was achieved in 83% of cases, which is similar to the previously reported GTR rate of 80–90% [2,3,5,6,14]. Factors preventing GTR may be diffuse extension within a nerve, tumor location, adhesion to the spinal cord due to inflammation, hemorrhage or subpial growth [5], or proximity to major blood vessels or joints. In fact, in this series, STR was more common in the cervical spine (50%) compared to the thoracic (11%) and lumbosacral spine (9%). This is arguably due to the intricate anatomy of the cervical spine and the close relationship to the vertebral artery. The aim of surgery in these cases was to alleviate spinal cord compression without aggressively pursuing extraforaminal tumor remnants. Subtotal resection increases the risk of tumor regrowth and is associated with a greater degree of postoperative neurological deficits [2,6,26]. In the study cohort, the frequency of tumor regrowth was significantly greater following STR (30%) than GTR (2%). In total, tumor growth was observed in nine of thirty STR cases; however, reoperation was only performed in five of these cases when the tumor exerted compression of the spinal cord or caused new symptoms such as pain. Asymptomatic tumor growths after STR were treated conservatively.

Schwannomas and meningiomas of the spine belong to the group intradural extramedullary tumors and are typically benign and slow-growing [27]. However, unlike meningiomas, which most commonly arise from the thoracic region, schwannomas show a clear predilection for lumbosacral segments where they can grow larger without compressing the spinal cord [28]. On imaging, schwannomas typically lack the dural tail sign, which is specific to meningiomas, but often adopt a dumbbell shape as they traverse the neural foramen [29]. Thus, while meningiomas generally cause myelopathic symptoms related to spinal cord compression, schwannomas may more commonly present with symptoms related to nerve root compression. In addition, schwannomas are typically soft tumors that exhibit cystic degeneration, as opposed to meningiomas which are commonly denser and associated with intratumoral calcifications [30].

Nonetheless, resection of spinal meningiomas is safe and effective; a previous report from the study center showed postoperative worsening in only 1.5% of cases, with the rest of the patients experiencing improvement or unchanged status in equal proportions [20]. Similarly, most patients with spinal schwannomas either improved or remained unchanged in neurological deficits. However, 7% of patients experienced a postoperative deterioration on the mMC. One explanation may be that even though the integrity of the nerve is prioritized, schwannoma surgery sometimes requires the sacrifice of the nerve to achieve GTR, and in cases of STR, juxtatumoral fibers may be damaged. In fact, some authors argue that sensory deficits are to be expected.

In this cohort of 180 surgically treated patients, only 6 received repeat surgical treatment for tumor recurrence (n = 1) after GTR or regrowth (n = 5) after STR. After renewed surgery, these patients reported an improved or unchanged mMC of I or II, indicating minimal to no symptoms. Nonetheless, there may be instances where non-surgical options are valuable. Radiotherapy has been suggested in poor surgical candidates or multiple tumors [9,31], and there have been reports indicating good local control of spinal nerve sheath tumors with the use of adjuvant radiotherapy [32,33,34]. Regardless, none of the 30 cases of STR in this cohort received adjuvant radiotherapy. Similarly, chemotherapy was not used and its role in the treatment of spinal schwannoma is currently limited to syndromic cases, which were absent from the current study cohort.

At the study center, radiological follow-ups were scheduled at 3 months and one year postoperatively. Additional imaging was performed upon further clinical findings and symptoms. All tumor recurrences or growths occurred within the first 4 years after surgery. As asymptomatic tumor recurrences or regrowths were as common as symptomatic ones, radiological follow-ups may allow early identification of cases that may potentially benefit from surgical treatment. However, based on the present findings, there seem to be no indication to extend radiological follow-ups beyond five years.

### Limitations

Despite the large cohort of patients with spinal schwannomas, the retrospective study design limits the aspects that can be evaluated. Other limitations include the follow-up period of 4.4 years, which may be too short to evaluate late recurrences after surgical resection. Neurophysiological monitoring was not used in many cases. The use of intraoperative neuromonitoring could provide a benefit for tumors with an anterior location or a plexiform growth since these cases may require more extensive manipulation of the spinal cord.

## 5. Conclusions

In this population-based cohort study, surgery was safe, with few complications, and resulted in significant improvements in neurological function at long-term follow-up. However, no predictive factors for postoperative improvement, for those with a preoperative neurological deficit, could be identified. The observation from this study, indicating that all instances of postoperative tumor progression occurred within 4 years, suggests the potential consideration of a final follow-up MRI at the 5-year mark.

## Figures and Tables

**Table 1:** Modified McCormick Scale.

Grade	Explanation
I	Intact neurologically, normal ambulation, minimal dysesthesia.
II	Mild motor or sensory deficit, functional independence.
III	Moderate deficit, limitation of function, independent with external aid.
IV	Severe motor or sensory deficit, limited function, dependent.
V	Paraplegia or quadriplegia, even with flickering movement.

**Table 2:** Baseline data.

Variables	Entire Cohort (n = 180)
Age (years)	53 (42–64)
Male sex	90 (50%)
Preoperative mMCs	
I	68 (38%)
II	92 (51%)
III	19 (11%)
IV	1 (0.6%)
Neurological deficits	
Motor deficit	74 (41%)
Sensory deficit	76 (42%)
Gait disturbance	32 (18%)
Bladder dysfunction	29 (16%)
Pain	156 (87%)
Tumor level	
Cervical	29 (16%)
Thoracic	70 (39%)
Lumbosacral	81 (45%)
Tumor location	
Intradural	146 (82%)
Extradural	12 (6.7%)
Combined	20 (11%)
Largest tumor area on axial plane (cm^2^)	1.4 (0.8–2.0)
Spinal cord tumors (C0–L2) (cm^2^)	1.5 (0.9–2.1)
Cauda tumors (cm^2^)	1.3 (0.7–1.9)
Spinal canal compression (%)	59 (35–70)
Spinal cord tumors (C0–L2)	60 (37–70)
Cauda tumors	53 (33–70)

Data presented as median (range) or count (proportion). Abbreviations: mMC = modified McCormick Scale; cm = centimeters.

**Table 3:** Surgical data.

Variables	Entire Cohort (n = 180)
Time from symptom presentation to surgery (months)	12 (6–24)
Time from diagnosis (first MRI) to surgery (months)	3 (1–6)
Laminectomy range (levels)	2 (2–3)
Laminoplasty	83 (46%)
Nerve root preserved	133 (74%)
Postoperative complication	
Wound infection requiring antibiotics	4 (2.2%)
Wound infection requiring surgery	0 (0%)
Hematoma requiring surgery	1 (0.6%)
CSF leak requiring surgery	5 (2.8%)
Gross total resection	150 (83%)
Adjuvant chemotherapy	0 (0%)
Adjuvant radiotherapy	0 (0%)

Data presented as median (range) or count (proportion). Abbreviation: CSF = cerebrospinal fluid.

**Table 4:** Clinical outcomes.

Variables	Entire Cohort (n = 180)
Radiological follow-up time (years)	1.7 (1.1–4.7)
Clinical follow-up time (years)	4.4 (2.0–8.3)
Postoperative mMCs	
I	121 (67%)
II	47 (26%)
III	12 (6.7%)
IV	0 (0%)
Tumor control	
Local recurrence following GTR	3/150 (2.0%)
Renewed surgery	1 (33%)
Watchful waiting	2 (67%)
Local growth following STR	9/30 (30%)
Renewed surgery	5 (56%)
Watchful waiting	4 (44%)
Dead	14 (7.8%)
Death due to tumor	0 (0%)

Data presented as median (range) or count (proportion).

**Table 5:** Change in neurological status at long-term follow-up.

	Motor Deficit	Sensory Deficit	Gait Disturbance	Bladder Dysfunction	Pain
Patients with preoperative deficit (n)	74 (41%)	76 (42%)	32 (18%)	29 (16%)	156 (87%)
Improved	67 (91%)	62 (82%)	29 (91%)	29 (100%)	130 (83%)
Completely improved	54 (81%)	46 (74%)	27(93%)	27(93%)	94 (72%)
Partially improved	13(19%)	16 (26%)	2 (7%)	2 (7%)	36 (28%)
Unchanged	6 (8.1%)	14 (18%)	2 (6.3%)	0 (0%)	22 (14%)
Worse (increased deficit)	1 (1.4%)	0 (0%)	0 (0%)	0 (0%)	4 (2.6%)
Worse (new deficit)	6 (6%)	10 (8%)	1(0.7%)	4 (3%)	4 (17%)
Postoperative improvement *p*-value (paired testing)	**<0.001**	**<0.001**	**<0.001**	**<0.001**	**<0.001**

Data presented as number (proportion). Bold text indicates a statistically significant correlation (*p* < 0.05).

**Table 6:** Univariate logistic regression predicting postoperative improvement in mMCs for those who were mMCs > I preoperatively.

Variable	OR (95% CI)	*p*-Value
Age (years)	0.99 (0.96–1.01)	0.404
Male sex	1.82 (0.84–4.03)	0.132
Intradural tumor	1.12 (0.34–7.01)	0.906
Cervical tumor	1.00 (0.34–3.15)	>0.999
Symptom duration (months)	1.00 (0.98–1.02)	0.951
Subtotal resection	0.83 (0.29–2.48)	0.734
Nerve root preservation	1.68 (0.81–3.48)	0.163
Tumor area for spinal cord tumors (C0–L2) (cm^2^)	0.71 (0.42–1.16)	0.179
Spinal cord compression for spinal cord tumors (C0–L2) (%)	0.61 (0.06–5.83)	0.669
Tumor area for cauda tumors (cm^2^)	0.63 (0.23–1.63)	0.343
Cauda compression for cauda tumors (%)	1.20 (0.03–41.6)	0.916

Data presented as median (range) or count (proportion). Bold text indicates a statistically significant correlation (*p* < 0.05).

## Data Availability

Upon reasonable request, the data may be provided by contacting the corresponding author, A.E.-T.
